# Handgrip strength testing in rheumatic diseases

**DOI:** 10.1007/s00296-026-06111-6

**Published:** 2026-04-29

**Authors:** Yuliya Fedorchenko, Umida Khojakulova, Olena Zimba, Burhan Fatih Kocyigit

**Affiliations:** 1 Department of Pathophysiology, Ivano-Frankivsk National Medical University, Ivano-Frankivsk, Ukraine; 2 Department of Emergency Medicine and Nursing, South Kazakhstan Medical Academy, Shymkent, Kazakhstan; 3 Department of Rheumatology, Immunology and Internal Medicine, University Hospital in Kraków, Kraków, Poland; 4 National Institute of Geriatrics, Rheumatology and Rehabilitation, Warsaw, Poland; 5 Department of Internal Medicine N2, Danylo Halytsky Lviv National Medical University, Lviv, Ukraine; 6 Department of Physical Medicine and Rehabilitation, University of Health Sciences, Adana City Research and Training Hospital, Adana, Türkiye

**Keywords:** Rheumatic diseases, Muscle strength, Handgrip strength, Rehabilitation, Digital health

## Abstract

Rheumatic diseases encompass diverse immune-mediated disorders that compromise musculoskeletal and systemic functions, often resulting in persistent disability. Hand muscle weakness is an early and clinically meaningful manifestation across rheumatoid arthritis (RA), systemic lupus erythematosus (SLE), and systemic sclerosis (SSc). Reduced handgrip strength (HGS) is an integrative measure that reflects systemic inflammation, neuromuscular involvement, and functional deterioration, providing critical insight into disease progression. The current review aims to overview available evidence on HGS testing in rheumatic diseases, with a focus on measurement devices, clinical and prognostic significance, and perspectives for its integration into disease monitoring and patient management. HGS is a sensitive marker of muscular function and frailty. Advances in mechanical and digital dynamometry, wearable devices, and smartphone-integrated systems enable precise and remote assessments. Reduced HGS correlates with higher disease burden and impaired quality of life. Despite its growing applicability, heterogeneity in testing procedures and inter-device variability underscore the need for standardized protocols and device-specific reference values. Advances in wearable sensors, digital dynamometry, and AI-supported telerehabilitation hold promise for integrating HGS into personalized disease monitoring.

## Introduction

Rheumatic diseases constitute a heterogeneous group of immune-mediated disorders that affect joints, muscles, nerves, vessels, and numerous other organ systems, manifesting with arthralgia, myalgia, weakness, and functional disability [1]. Despite advances in immunotherapy and disease-modifying treatments, persistent functional impairment remains a major clinical challenge [2]. Muscle weakness, particularly in the hands, is an early and long-lasting manifestation across rheumatic diseases [3].

In recent years, HGS has received growing attention as a proxy for overall health status, with accumulating evidence indicating its value in predicting disability, frailty, sarcopenia, and mortality in the general population [4, 5]. HGS isa sensitive measure of rheumatic disease activity, reflecting both inflammatory burden and functional capacity [6, 7].

HGS studies include patients with rheumatoid arthritis (RA), systemic lupus erythematosus (SLE), and systemic sclerosis (SSc) who present with compromised hand functions due to inflammation, pain, muscle wasting, and skin disorders [8–10]. Available evidence suggests that patients with RA display much worse HGS performance compared to those with SLE, pointing to structural damage and functional impairment associated with rheumatoid hand [11]. HGS may reflect accelerated aging in SLE [12] and skin thickening and loss of dexterity in SSc [10, 13].

Advances in dynamometry have expanded the domain of HGS assessment, evolving from mechanical devices to digital, sensor-based, and smartphone-integrated systems, facilitating more precise and reproducible evaluations of grip performance [14]. These technological innovations not only improve measurement accuracy but also facilitate remote tracking, enabling early detection of functional deterioration and prompt application of individualized treatment measures [14].

This review aims to overview current evidence on HGS testing in rheumatic diseases, focusing on measurement devices, clinical and prognostic values, and perspectives for its integration into disease monitoring and patient management.

### Search strategy

Comprehensive searches were conducted via Medline/PubMed, Scopus, and the Directory of Open Access Journals (DOAJ) to identify reports on HGS testing, assessment devices, monitoring values, and clinical implications in patients with rheumatic diseases. The analyses included systematic reviews, clinical trials, observational studies, case series, and case reports published up to November 1, 2025. Search terms included combinations of disease and outcome-related keywords using Boolean operators: ("rheumatoid arthritis" OR "systemic lupus erythematosus" OR "systemic sclerosis" OR "spondyloarthritis” OR “osteoarthritis” OR “inflammatory arthritis” OR “autoimmune rheumatic disease”) AND (“hand grip strength” OR “hand grip test” OR “dynamometer” OR “hand function” OR “grip assessment” OR “muscle strength”) AND ("device" OR "instrument" OR "measurement" OR "monitoring" OR "clinical evaluation" OR "functional assessment" OR "disease activity"). Only English articles were included in the analyses. The search and selection of articles followed widely publicized recommendations for comprehensive bibliographic searches [15].

## Devices for handgrip strength measurements

HGS reflects the integrated performance of skeletal muscles, tendons, joints, and neuromuscular structures [4]. The accurate measurements are essential for monitoring disease progression, evaluating treatment response, and guiding rehabilitation strategies [16]. Available devices include mechanical and digital dynamometers, each with distinct advantages and limitations [17].

The Jamar® hydraulic dynamometer is considered the gold standard for HGS measurements [18], offering five adjustable handle positions with the second position recommended by the American Society of Hand Therapists (ASHT) [19]. Its 1.5 kg weight and firm metal handles may pose challenges for some patients with arthritis and osteoporosis, for whom lighter pneumatic alternatives are preferable [20]. Smedley dynamometers, such as Baseline and Takei, are widely used for their reliability and adaptability to various hand sizes [20].

In a cross-sectional study of 121 individuals with RA and matched controls, HGS and pinch strength, measured using Jamar® devices, showed substantial deficits in RA patients and correlated with rheumatoid activity and functional indices (e.g., HAQ) [21]. These findings confirm that mechanical dynamometry provides reproducible measures of functional impairments [21].

Inter-device comparisons reveal that measurement variability remains a significant issue [19]. When tested in healthy adults, hydraulic devices tend to yield handgrip values approximately 10% higher than those from electronic dynamometers [22]. Therefore, consistency in the choice of device for longitudinal assessments is critical to ensure data comparability.

Overall, current HGS measurement devices can be categorized into hydraulic and electronic dynamometers, distinguished by their sensing mechanisms. Hydraulic models employ a load cell integrated into a movable handle that compresses against a fixed element, translating mechanical pressure into a measurable force. By contrast, electronic systems use force-sensitive resistors (FSRs) to detect impedance variations induced by handgrip pressure, providing high-resolution data [17]. The integration of these digital technologies into sensorized gloves and ergonomic modules marks a transition toward accurate assessments, enabling more precise quantification and facilitating remote monitoring of hand functions [17].

**Fig. 1:**
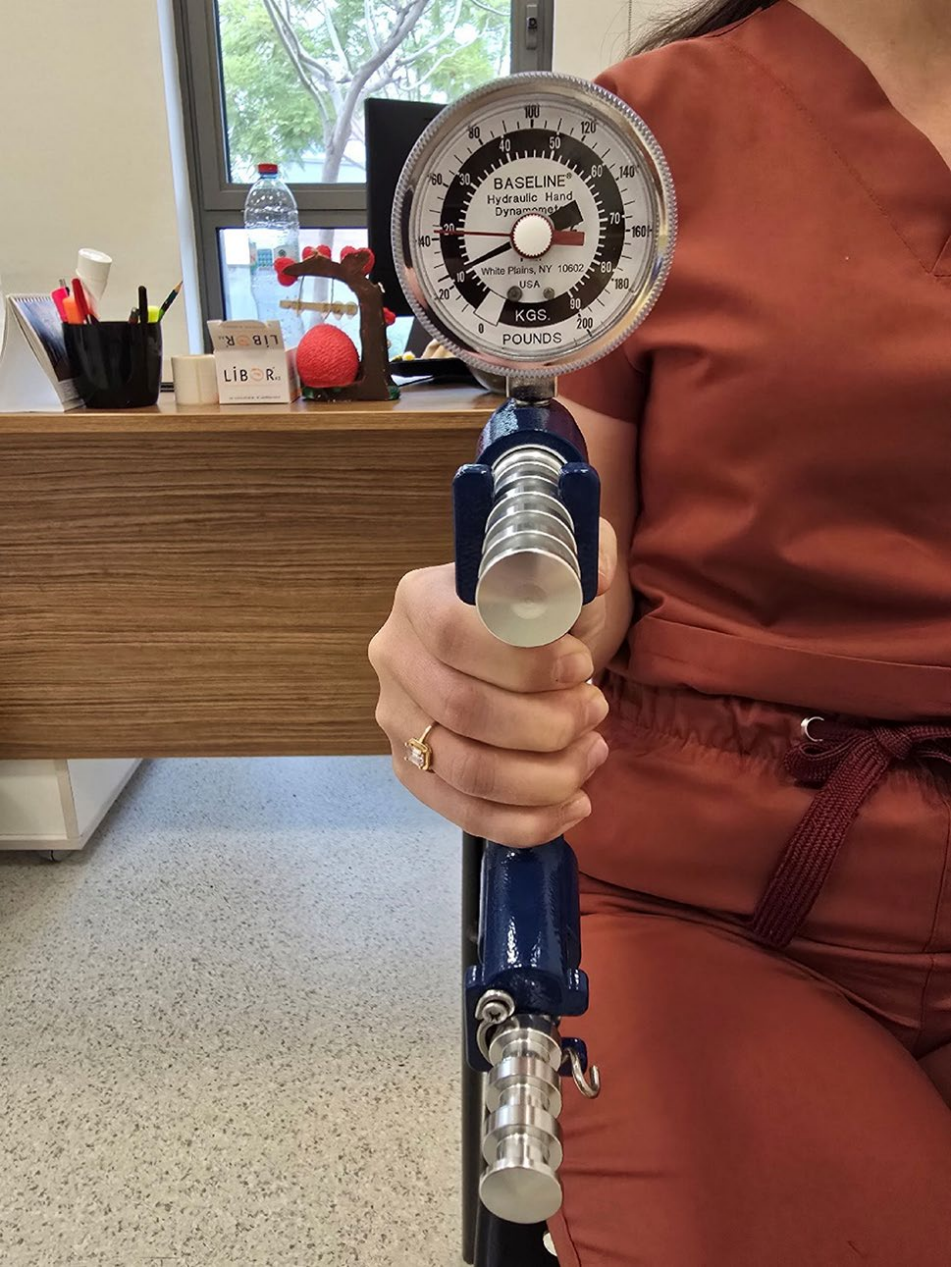
Baseline Hydraulic Hand Dynamometer—Standard Testing Position (Front View). The participant demonstrates the recommended testing posture using a Baseline® hydraulic hand dynamometer. The device is shown from the front, with the wrist in a neutral position and the elbow supported at approximately 90°, consistent with ASHT testing guidelines

**Fig. 2:**
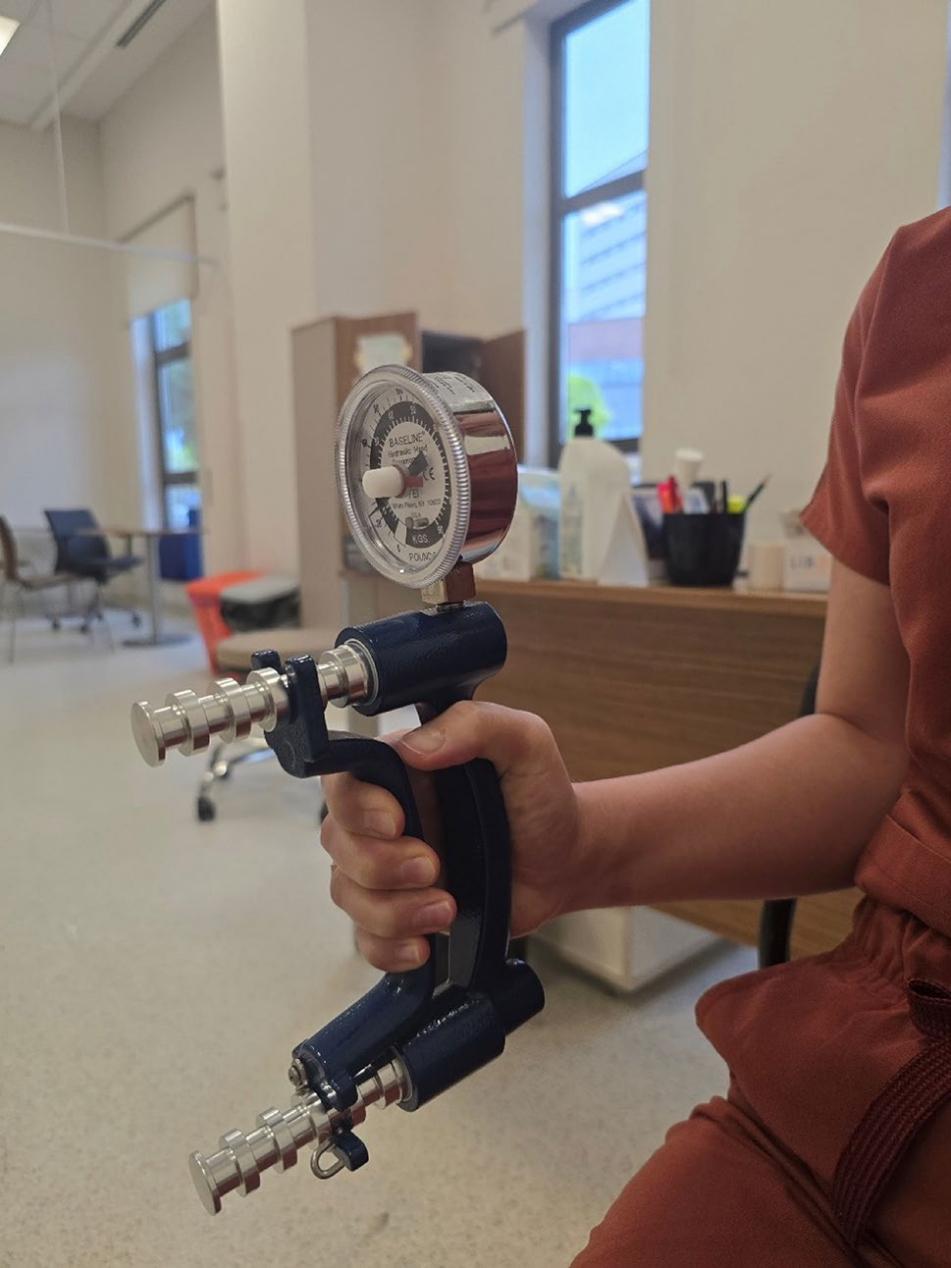
Baseline Hydraulic Hand Dynamometer—Grip Application (Side View). Side view illustrating proper grip technique during maximal voluntary contraction. The participant’s shoulder is adducted, elbow stabilized, and the handle of the hydraulic dynamometer is set to the standardized testing position

Unlike hydraulic models, digital dynamometers capture the full force–time curve, allowing evaluation of not only maximal isometric force but also dynamic parameters such as rate of force development and fatigue resistance [23]. This multidimensional approach enables the detection of subtle neuromuscular deficits that may precede overt functional decline [23]. Correct testing posture using a hydraulic dynamometer is shown in Figs. 1 and 2.

Jamar PLUS + Digital and Takei TKK 5401 dynamometers demonstrate high validity, though they tend to slightly overestimate HGS relative to hydraulic systems, underscoring the need for device-specific reference values [24].

Among commercially available devices, the CAMRY EH101 digital dynamometer has shown strong agreement with the Jamar® hydraulic standard and minimal bias [25]. Comparative studies demonstrate excellent test–retest reliability for both new and heavily used units, with negligible inter-device variation compared with the Takei TKK 5401 (0.8 kg) [26]. These findings indicate that CAMRY EH101 is a precise alternative for clinical and research applications [25, 26].

In a cohort of 1,064 community-dwelling adults aged 50–90 years, HGS values measured with the CAMRY were identical to those measured with the Jamar device, with minimal gender differences (0.5 kg for men and 0.6 kg for women) [27]. Regression modeling demonstrated a consistent, predictable relationship between the two instruments, distinguishing CAMRY EH101 as a valid proxy for Jamar® in both clinical and epidemiological contexts [27, 28].

Digital dynamometry has evolved toward integration with interactive rehabilitation systems. The Pablo Tyromotion platform exemplifies this innovation, combining virtual reality environments with biofeedback-based HGS measurements [29]. This system demonstrates excellent reliability, and it enhances patient motivation through gamified, task-specific training [29]. Such advances distinguish digital HGS measurements as diagnostically important.

Recent technological advances have enabled patient-centered, real-time HGS measurements. Smartphone-integrated and wearable systems provide continuous, objective measurements of hand function, enabling early detection of functional decline and personalizing therapeutic interventions. In a study on 82 RA patients, smartphone-integrated dynamometry showed a significant inverse correlation between HGS and DAS28 (r = – 0.65; *p* < 0.001) [30].

Several mobile systems have been designed to monitor hand functions in older adults. The Eforto® system—an integrated, smartphone-connected platform—enables quantification of hand muscle fatigue, including fatigue resistance (time to 50% maximal HGS) and handgrip work (area under the force–time curve) [31]. In a cohort of 112 community-dwelling adults and geriatric inpatients, Eforto® showed acceptable accuracy of HGS measurements compared with the Martin Vigorimeter (*r* = 0.95), fatigue resistance (*r* = 0.81), and grip work (*r* = 0.73) [31]. Reliability analysis showed moderate-to-excellent intra- and inter-rater agreement (ICC = 0.59–0.94), indicating that Eforto® is a reliable instrument for clinical and home-based monitoring of hand performance [31].

Smartphone-based systems have been designed to enable ongoing home-based monitoring as part of telerehabilitation. One such model combines the eGripper device with the grippyBird gamified platform and clinician dashboard for remote supervision [32]. Based on principles of neuroplasticity, this system delivers repetitive, task-specific exercises to enhance HGS. Validation studies reported high reliability and strong agreement with the Jamar® dynamometer [32].

**Fig. 3:**
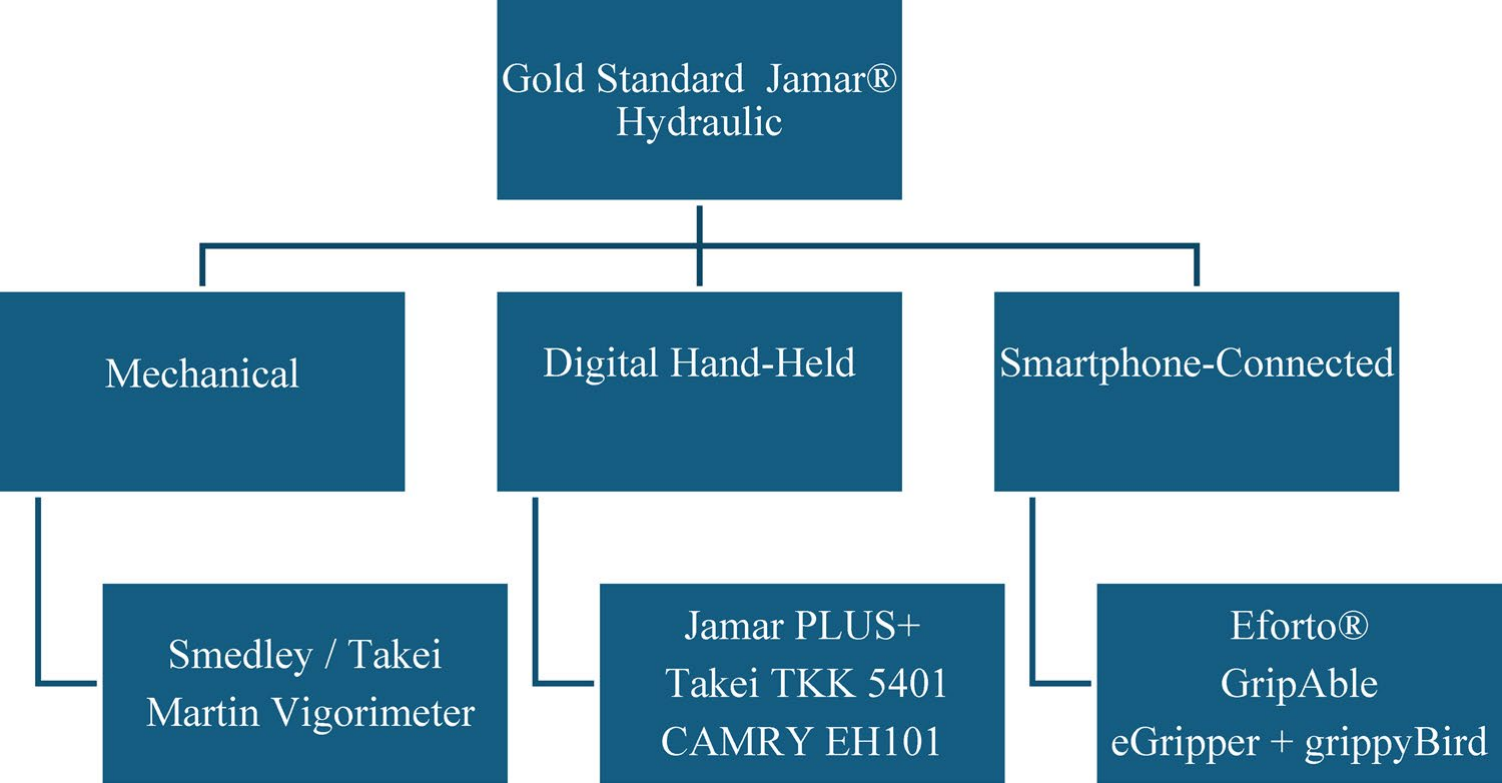
Overview of Handgrip Strength Dynamometers Used in Rheumatic Diseases

GripAble is another innovative handgrip device for wireless measurement of isometric and elastically resisted grip forces. Laboratory testing demonstrated high accuracy (error < 1.67 kg) and sensitivity (0.062 ± 0.015 kg), comparable to or exceeding the Jamar® standard [33]. GripAble demonstrated excellent test–retest reliability (ICC = 0.971–0.975) and strong inter-device agreement (ICC = 0.898–0.922) [34]. GripAble’s high reliability and user-friendly interface make it a valuable tool for remote monitoring [33–35]. An overview of the commonly used devices in rheumatic diseases is presented in Fig. 3.

### Standardization of handgrip strength measurements

Despite the availability of standardized guidelines from the ASHT [36], variability of HGS measurements persists. The variability stems from differences in contraction duration, scoring approaches, and procedural techniques, leading to irreproducible research and clinical outcomes [37]. HGS training and clinical experience confound adherence to the ASHT protocol and the interpretation of the results obtained [16]. As such, adopting a unified, standardized HGS testing protocol is warranted to improve the reproducibility and cross-study comparability of measurements [16].

### Handgrip strength in rheumatoid arthritis

HGS represents a sensitive and reliable indicator of muscular function, functional independence, and disease burden in RA [6]. Case–control studies reveal markedly lower HGS in RA, with notable gender differences [8, 21]. In a cohort of 100 RA patients and 100 matched controls, both male and female patients demonstrated significantly reduced HGS, with men showing a disproportionately greater HGS decline (right hand F₁,₁₉₅ = 14.62 kg, *p* < 0.01; left hand F₁,₁₉₅ = 20.54 kg, *p* < 0.01) [8]. Large-scale Korean population data (n =13,966) further confirm the association between decreased HGS and lower quality of life, particularly in the domains of mobility, self-care, daily activities, and pain/discomfort [38].

A 24-week randomized trial (n = 106) supports exercise as the key modifiable factor for improving HGS in RA [39]. A 24-week dynamic exercise program (DEP) produced the greatest HGS gains (median + 2 kg) compared with combined DEP–Mediterranean diet (MD; + 0.5 kg) or MD alone (− 0.5 kg; *p* = 0.03) [39].

Innovative approaches to HGS measurements, such as cylindrical grip devices that capture force–time parameters, have demonstrated strong correlations with disease activity and anthropometric factors, validating their clinical utility as integrative markers of functional impairment (ROC AUC = 0.81, 95% CI 0.746–0.864) [6].

HGS is an indicator of frailty in RA [40]. Also, reduced HGS in RA may reflect impaired vascular function presenting with concurrent muscular and circulatory deficits [41]. Finally, HGS may reflect patients' employability and work ability [42].

HGS testing is mostly informative in early inflammatory arthritis [43]. In a systematic review of 27 observational studies on 2,742 patients with RA, HGS showed a modest overall improvement over time [43]. Improvements were pronounced in early RA, whereas no significant change was observed in established disease [43].

### Handgrip strength in lupus

HGS is an indicator of muscular performance, cardiometabolic health, and functional capacity in lupus [44]. A systematic review demonstrated that HGS in patients with lupus was significantly lower than in healthy controls (SLE = 21.7 kg vs. controls = 29.3 kg; *p* < 0.05), with the lowest values reported in patients with deforming arthropathy [9]. In line with these findings, premenopausal women with low lupus activity displayed marked deficits in both handgrip and dynamic muscle strength compared with matched healthy women (leg press − 25.6%, chest press − 18.3%, lat pulldown − 13.6%; all *p* < 0.05) [45].

Cross-sectional studies link higher HGS to superior quality of life across physical domains of the SF-36 [46]. HGS also correlates with favorable body composition and cardiometabolic profiles in SLE [44]. HGS was independently associated with lower adiposity indices, including BMI, fat mass, and waist-to-height ratio in SLE [47]. Importantly, low HGS was reported as an early sign of sarcopenia and related functional decline in SLE [48]. Overall, HGS can be viewed as a sensitive integrative marker of muscular, metabolic, and cardiovascular health in patients with SLE.

### Handgrip strength in systemic sclerosis

HGS is a robust indicator of overall muscle function in SSc, reflecting both peripheral muscle involvement and general physical capacity [49]. In a cohort of 26 women with SSc, HGS was markedly reduced compared with healthy controls and correlated with decreased knee muscle strength and higher fatigue and disability [49]. Individuals with SSc and sarcopenia demonstrated significantly reduced HGS, decreased knee extension strength, and lower SF-36 physical function scores [50].

HGS can be a valuable indicator of the efficiency of therapeutic interventions in SSc aimed at improving muscular and physical function [51]. In fact, a 12-week high-intensity interval training (HIIT) program significantly increased HGS, inspiratory muscle strength, and walking speed [51]. A systematic review encompassing 26 studies and 2,661 participants with immune-mediated rheumatic diseases revealed that women had substantially lower HGS compared with healthy controls, with weakness correlating with disease duration, activity, and functional limitation in SSc, RA, and SLE [52].

Further supporting the systemic nature of muscle impairment, SSc patients demonstrate concurrent reductions in peripheral and respiratory muscle strength [53]. In a study on 16 SSc patients and matched controls, HGS moderately correlated with quadriceps thickness (*ρ* = 0.576; *p* = 0.02) [50]. Handgrip performance is further linked to disability outcomes [54]. In 28 SSc patients, isometric HGS and pulmonary function were inversely correlated with Health Assessment Questionnaire Disability Index (HAQ-DI) scores [54].

Functional assessments, such as the TGlittre-Shelf (TGlittre-S) test, reinforce the role of hand strength in daily activity performance in SSc [13]. Among 41 women with SSc, hand function measured by TGlittre-S was significantly impaired compared to controls, negatively correlating with HGS (rs =  − 0.511; *p* = 0.0006) and positively correlating with disability (HAQ-DI, rs = 0.510; *p* = 0.0006) [13].

Improving hand function may translate into broader gains in physical performance in SSc [55]. A 24-week supervised physiotherapy and occupational therapy program with home exercises in 59 patients yielded sustained improvements in HGS (Δ =  + 3.2 kg; *p* < 0.01), hand and mouth function, and disability scores [55]. The gains exceeding 20% persisted at week 48, underscoring the durability of rehabilitation benefits and the importance of integrated, supervised therapy for maintaining muscle strength and functional capacity in SSc [55].

### Handgrip strength in psoriatic arthritis

Psoriatic arthritis (PsA) is a chronic inflammatory disease with multisystem involvement that leads to substantial functional limitations and negative psychosocial consequences [56]. Evidence from systematic reviews underscores the relevance of muscular dysfunction in PsA, showing that although resistance training in active disease does not significantly increase muscle strength, it yields meaningful improvements in functional capacity, disease activity, pain, and overall health status [57]. Handgrip strength in individuals with PsA is reduced compared with healthy controls [58, 59].

Reduced HGS and impaired hand function in PsA are comparable to the same deficits in RA [60, 61]. Importantly, prospective interventional data suggest that HGS remains stable during substantial weight loss in obese patients with PsA, despite reductions in lean mass, indicating relative preservation of upper-limb strength under metabolic stress [62].

### Disease-specific mechanisms affecting handgrip strength

HGS in rheumatic disorders reflects not only systemic inflammation and overall neuromuscular involvement but also disease-specific pathogenic mechanisms. RA involves joint deterioration, synovitis, and tendon issues, leading to pain, stiffness, and reduced hand mobility, all of which directly affect HGS. In SLE, myositis, chronic inflammation, and accelerated sarcopenia lead to reduced muscle strength and functional ability [9, 11, 21]. SSc is characterized by skin fibrosis, digital contractures, and microvascular alterations, all of which impair hand function, dexterity, and coordination [13]. In PsA, entheseal inflammation, synovitis, and joint deterioration, along with soft tissue and tendon issues, impair hand function and weaken grip [59]. Recognizing these disease-specific mechanisms is essential for accurately interpreting HGS results and developing targeted rehabilitation and treatment strategies.

### Handgrip strength from a rehabilitation perspective

HGS is crucial in the rehabilitation of patients with rheumatic diseases, acting as both a diagnostic marker and a treatment target. HGS may reflect the integrity of neuromuscular function, joint mobility, soft-tissue flexibility, and functionality of the upper extremity. HGS decline may indicate compromised daily functioning and heightened frailty, underscoring the need to evaluate it when developing patient-centered rehabilitation strategies [63, 64].

Novel technologies, including digital dynamometry, virtual reality training platforms, and telerehabilitation tools, enhance rehabilitation by allowing accurate monitoring of grip performance, delivering real-time biofeedback, and promoting compliance with home-based therapies [29, 65]. In this context, HGS serves as an objective indicator that informs clinical decision-making, monitors therapeutic dynamics, and facilitates individualization of rehabilitation strategies.

### Assessment of digital and wearable technologies in patient groups

Recent advances in digital dynamometry, wearable sensors, and smartphone-integrated systems have enhanced the abilities of HGS evaluation, providing accurate, continuous, and remote monitoring. Although these technologies have considerable potential, their validation, practicality, and constraints within rheumatic populations warrant further evaluation [30]. The device's accuracy may fluctuate due to hand deformities, joint pain, or impaired mobility, which are common in rheumatic disorders [66]. Furthermore, patient adherence, ease of use, and technical literacy may affect the reliability of home-based or telerehabilitation measurements. Understanding these factors is crucial for interpreting HGS data, designing rehabilitation programs, and guiding future research on remote or digital assessments.

### Future directions

Further research on HGS in rheumatic diseases should prioritize enhancing methodological consistency and device interoperability. Defining disease-specific thresholds for functional deterioration and clinically important changes may enhance the accuracy of HGS measurements for monitoring and decision-making.

Emerging wearable sensors, time–force curve analysis, machine learning prediction models, and AI-powered telerehabilitation methods offer opportunities to improve the precision and clinical applicability of grip strength evaluations. Further studies should explore integrating these methods into remote-monitoring systems to facilitate prompt identification of functional decline.

## Conclusions

HGS measurements reflect global muscle function, disease activity, and functional capacity in RA, SLE, SSc, and PsA. Reduced HGS correlates with higher disease burden, frailty, impaired physical performance, and diminished quality of life.

Advances in digital and smartphone-based dynamometry enable precise, remote monitoring, facilitating early detection of functional decline and guiding personalized interventions. Structured rehabilitation programs, including guided home exercises, improve HGS, daily functioning, and overall physical performance. Standardized measurements of HGS are essential to ensure reproducible, comparable results. Taken together, HGS is a valuable marker for disease monitoring, therapeutic guidance, and rehabilitation in rheumatic diseases.
